# Supplementary material for: A robust algorithmic cum integrated approach of interval-valued fuzzy hypersoft set and OOPCS for real estate pursuit
Source: PeerJ Comput Sci. 2023 Jun 22;9:e1423. doi: 10.7717/peerj-cs.1423 (PMC10319272; doi:10.7717/peerj-cs.1423)
Supplement: Supplemental Information 1 [file peerj-cs-09-1423-s001.docx]

Raw Data

**Manuscript Title: A robust algorithmic cum integrated approach of interval-valued fuzzy hypersoft set and OOPCS for real estate pursuit**

Following are the sources for the raw considered in the above-mentioned manuscript:

1. **Parameters and their respective sub parametric values (Source: Literature review)**
2. Location
3. Price
4. Plot size
5. Adaptation to weather
6. Covered area
7. Number of bed room
8. Access to main road
9. Number of bath rooms
10. Number of floor
11. Green surrounding
12. Recreation facility
13. Educational Institution
14. Health Facility
15. **Linguistic term for evaluation of parameters**

| Linguistic Term | Interval valued Fuzzy value |
| --- | --- |
| Extremely important | [ 0.86,1.00 ] |
| Very important | [ 0.66,0.85 ] |
| Important | [ 0.36,0.65 ] |
| Un-important | [ 0.16,0.35 ] |
| Irrelevant | [ 0.00,0.15 ] |

1. **Parameters, their description and measuring units**

| Attribute | Description | Measuring units |
| --- | --- | --- |
| Location | Non-Ideal (not main city), ideal (main city) | - |
| Price | American dollars | million dollars |
| Plot size | Dimensions/area of plot | square meter |
| Adaptation to weather | Design of house in accordance with climate | - |
| Covered area | Area covered by infrastructure (building) | part of total area |
| Bed rooms | Rooms with at-least 1 window | Number |
| Access to main road | Distance from main road/highway | Kilometer |
| Bath rooms | Including bathrooms attached to bedrooms | Number |
| Floor | Single story, multi-story | Number |
| Green surrounding | Public park nearby | - |
| Recreation facility | Cinema, zoo nearby | - |
| Educational Institution | Distance | meter |
| Health Facility | Public hospital nearby | meter |
| Security and safety system | Minimum time for arrival of emergency services | minutes |
| Market | Distance | Meter |
| Architecture | Material used | - |

1. **Parameters, their sub parameters, respective sub parametric values and fuzzy valued intervals**

| Attribute | Sub Attribute | Prescribed value | Fuzzy Value |
| --- | --- | --- | --- |
| Location | non-ideal, ideal | 0,1 | [ 0,0.50] , [ 0.51,1] |
| Price | Less than or equal to 0.1 M dollar, greater than 0.1 M dollar up to  0.15 M dollars, greater than 0.15 M dollars | 0 ≤ p ≤ 0.1, 0.1 <  p ≤ 0.15, p > 0.15 | [ 0,0.33], [0.34,0.66], [0.67,1] |
| Plot size | less than or equal to 300m^2^ , greater than 300m^2^ | 0 ≤ s ≤ 300, s > 300 | [0, 0.50], [0.51, 1] |
| Adaptation to weather | extremely hot climate (EH), extremely cold climate (EC), EH&EC | 1,2,3 | [ 0,0.33], [0.34,0.66], [0.67,1] |
| Covered area | less than or equal to 50%, greater than 50% | 1,2 | [0, 0.50], [0.51, 1] |
| Bed rooms | less than 3, 3 to 5, greater than 5 | 2,5,6 | [ 0,0.33], [0.34,0.66], [0.67,1] |
| Access to main road | main road, less than 2km, greater than 2km | 0,1,2 | [ 0,0.33], [0.34,0.66], [0.67,1] |
| Bath rooms | less than 3, 3 to 4, more than 4 | 2,4,5 | [ 0,0.33], [0.34,0.66], [0.67,1] |
| Floor | 1, 2, 3, more than 3 | 0,1,2,3 | [0, 0.25], [0.26, 0.50], [0.51, 0.75], [0.76, 1] |
| Green surrounding | available / not available | 0,1 | [0, 0.50], [0.51, 1] |
| Recreation facility | available / not available | 0,1 | [0, 0.50], [0.51, 1] |
| Educational Institution | less than or equal to 300m, more than 300m but less than 1km, more than 1km | 0,1,2 | [0, 0.33], [0.34, 0.66], [0.67, 1] |
| Health Facility | less than or equal to 500m, more than 500m | 1,2 | [0, 0.50], [0.51, 1] |
| Security and safety system | less than or equal to 5min, greater than 5min | 1,2 | [0, 0.50], [0.51, 1] |
| Market | less than or equal to 500m, greater than 500m | 1,2 | [0, 0.50], [0.51, 1] |
| Architecture | concrete, wood | 1,2 | [0, 0.50], [0.51, 1] |

1. **Softwares**
2. Microsoft Excel 2010
3. Microsoft Word 2010
4. WinEdt version Build: 20121130 (v. 7.0)
5. MathType version 6.9
