# Supplementary material for: A robust algorithmic cum integrated approach of interval-valued fuzzy hypersoft set and OOPCS for real estate pursuit
Source: PeerJ Comput Sci. 2023 Jun 22;9:e1423. doi: 10.7717/peerj-cs.1423 (PMC10319272; doi:10.7717/peerj-cs.1423)
Supplement: Supplemental Information 2 [file peerj-cs-09-1423-s002.docx]

**Code**

**Manuscript Title: A robust algorithmic cum integrated approach of interval-valued fuzzy hypersoft set and OOPCS for real estate pursuit**

In the above-mentioned manuscript, the authors have not used any machine learning tools or computer languages based softwares therefore there is no coding involved in the manuscript to design the algorithm. However, the methodological steps, without coding indices, in general template are being presented below that can be executed by any machine learning tool after transformation in codes:

1. Statement of the problem: Consider as the set of decision makers, be the alternatives and $X=\left\{x_{1},x_{2},...,x_{n}\right\}$ be the set of parameters. Then a hypersoft set can be defined as where such that each attribute $x_{1},x_{2},...,x_{n}$ corresponds to a unique disjoint attribute valued set $X_{1},X_{2},...,X_{n}$ Let then each is $n-$tuple element.
2. Construction of weighted interval valued fuzzy parameter hypersoft set represented in the form of matrix $\mathbf{M}_{\mathbf{D}}$ displayed where is linguistic rating assigned by decision maker $\Delta_{i},$ the sub-parametric tuple
3. Calculation of mean difference of each interval of weighted interval-valued fuzzy parameter matrix $\mathbf{M}_{\mathbf{D}}$ by $x_{t}=\frac{b_{t}-a_{t}}{2}$ obtained for each interval $(a_{t},b_{t})\in\mathbf{M}_{\mathbf{D}}$
4. Construction of weighted vector $\mathbf{W}=\left\{\mathbf{W}_{1},\mathbf{W}_{2},...,\mathbf{W}_{n}\right\}.$ The elements of weighted vector $\mathbf{W}$ can be calculated by utilizing
5. Construction of fuzzy decision matrix $\mathbf{D}_{k}$ displayed corresponding to each decision maker $\Delta_{k}$
6. Construction of average interval-valued fuzzy hypersoft decision matrix $\mathbf{V}$ using equation $\mathbf{V}=\frac{1}{n}\left(\mathbf{D}_{1}\oplus\mathbf{D}_{2}\oplus...\oplus\mathbf{D}_{n}\right)=\left[\zeta_{ij}\right]_{m\times n}$ $\left[\zeta_{ij}\right]_{m\times n}=\left[\frac{\zeta_{ij}^{l1}+\zeta_{ij}^{l2}+...+\zeta_{ij}^{ln}}{n},\frac{\zeta_{ij}^{u1}+\zeta_{ij}^{u2}+...+\zeta_{ij}^{un}}{n}\right]_{m\times n}$ where represent matrices sum taken for corresponding lower bonds $\zeta^{l1},\zeta^{l2},...,\zeta^{ln}$ and corresponding upper bonds $\zeta^{u1},\zeta^{u2},...,\zeta^{un}$ of interval respectively.
7. Construction of Mean difference of average interval-valued fuzzy parameter matrix by $x=\frac{b-a}{2}$ for interval $(a,b)$
8. Construction of weighted fuzzy decision matrix $\mathbf{V}_{\mathbf{W}}$
9. Figuring fuzzy-valued $+ive$ ideal solution $(^{+}IS)$ and fuzzy-valued $-ive$ ideal solution $(^{-}IS).$ They are obtained with the help of fuzzy set theory and the TOPSIS technique;

1. Figuring separation measures $(\mathbf{SM}_{i}^{+})$ and $(\mathbf{SM}_{i}^{-})$ for each parameter.
2. Calculation of the nearness of alternatives to the optimal solution $\mathbf{RC}_{i}^{+}$

$$\mathbf{RC}_{i}^{+}=\frac{\mathbf{SM}_{i}^{-}}{\mathbf{SM}_{i}^{-}+\mathbf{SM}_{i}^{+}},0\leq\mathbf{RC}_{i}^{+}\leq1,\forall i\in\aleph_{m}$$

1. Ranking the preference order.
